# Hyperactivity persists in male and female adults with ADHD and remains a highly discriminative feature of the disorder: a case-control study

**DOI:** 10.1186/1471-244X-12-190

**Published:** 2012-11-07

**Authors:** Martin H Teicher, Ann Polcari, Nikolaos Fourligas, Gordana Vitaliano, Carryl P Navalta

**Affiliations:** 1 Developmental Biopsychiatry Research Program, McLean Hospital, 115 Mill Street, Belmont, MA, 02478, USA; 2 Department of Psychiatry, Harvard Medical School, Boston, MA, USA; 3 School of Nursing, Northeastern University, Boston, MA, USA; 4 Department of Biomedical Engineering, Tufts University, Medford, MA, USA; 5 Mental Health Counseling & Behavioral Medicine Program, Boston University School of Medicine, Boston, MA, USA

**Keywords:** ADHD, Attention, Hyperactivity, Impulsivity, Laboratory Tests, Biomarkers, Receiver Operating Characteristic, Executive Functions

## Abstract

**Background:**

Symptoms of hyperactivity are believed to fade with age leaving ADHD adults mostly inattentive and impulsive. Our aim was to test this assertion using objective measures of hyperactivity, impulsivity and inattention.

**Method:**

Participants were 40 subjects with ADHD (23M/17F; 35±10 yrs) and 60 healthy adults (28M/32F; 29±9 yrs) blindly assessed using Wender-Reimherr interview ratings, Structured Clinical Interview for DSM-IV Disorders and DSM-IV criteria. Infrared motion capture systems tracked head and leg movements during performance of a No-4’s cognitive control task. Subjects also completed the Conners’ CPT-II.

**Results:**

ADHD and controls differed significantly in activity and attention. Effect sizes for activity measures (d’ = 0.7–1.6) were, on average, two-fold larger than differences in attention or impulsivity, correlated more strongly with executive function ratings and were more discriminatory (ROC area = 0.83 for activity composite, 0.65 for No-4’s distraction composite, 0.63 for Conners’ CPT-II confidence index, 0.96 for the combined activity and attention diagnostic index). This finding was true for subjects with the predominantly inattentive subtype as well as subjects with combined or predominantly hyperactive/impulsive subtype. Males and females with ADHD were equally active. The superior accuracy of activity measures was confirmed using Random Forest and predictive modeling techniques.

**Conclusions:**

Objectively measured hyperactivity persists in adults with ADHD and is a more discriminative feature of the disorder than computerized measures of inattention or impulsivity. This finding supports the hypothesis that a deficient ability to sit still remains a defining feature of the disorder in adults when it is measured objectively.

## Background

Attention-Deficit/Hyperactivity Disorder (ADHD) is defined by signs of inattention, hyperactivity and impulsivity [1]. However, a widely held belief exists that hyperactivity fades with age or becomes merely a subjective sensation, leaving adults with ADHD to manifest primarily inattentive and impulsive symptoms [1]. This observation is interesting as it suggests that neural maturational events occur in individuals with ADHD that predominantly normalize motor activity but fail to provide the same degree of improvement in other symptoms. This view however, differs substantially from Wender’s initial description of the syndrome in adults, and from the criteria he developed [2]. The goal of this study was to test the hypothesis that hyperactivity is a less robust feature of ADHD in adults than inattention.

Hyperactivity is a sign rather than a symptom [3] and can be quantified using instruments. Porrino et al. [4] were the first to prove that youngsters with DSM-III Attention Deficit Disorder with Hyperactivity had higher activity levels than typically developing children. Teicher et al. [5] found that the seated hyperactivity of children with ADHD could be precisely quantified using infrared motion analysis to track the position of reflective markers during performance of a computerized attention task. Infrared motion analysis tracks movements with great accuracy and is employed by the movie industry to capture the action and facial expressions of actors to realistically animate computer-generated characters. Using this technology we reported that boys with ADHD moved their head 2.3 times more often than normal children, moved 3.4 times as far and had a more linear and less complex movement pattern [5]. Levels of hyperactivity correlated with an indirect measure of blood flow in the putamen [6] and with dopamine D2 receptor density in the caudate [7].

The aim of this study was to compare head and lower extremity movements of adults with ADHD to healthy controls. If hyperactivity abates then there should be little difference between adults with ADHD and healthy adult controls. If hyperactivity fades to a disproportionately greater degree then differences between ADHD and controls on motion measures should be less robust than differences in attention.

## Methods

### Participants

Subjects were 100 adults in good physical health, between 18–57 years of age, recruited from the community by advertisements; with a primary diagnosis of ADHD, or were healthy unmedicated controls without psychiatric disorders. Exclusion criteria included any use of drugs, herbal remedies, or non-ADHD medications (except contraceptives) for at least 2 weeks prior to enrollment. Subjects receiving treatment for ADHD were excluded if treated with anything other than short-acting stimulants. Further, they needed to be willing to stop stimulants for at least 24 hours prior to each visit. Additional exclusion criteria included a history of any major medical or neurological disorder that could affect motor activity or attention; major depression, bipolar disorder or anxiety disorders within the past 6 months; or any past or present history of alcohol or substance abuse.

### Protocol

This was a two-visit study approved by the McLean Hospital IRB. The study was explained on the first visit and written informed consent obtained from all participants. Diagnostic assessments were made using DSM-IV criteria and the Structured Clinical Interview for DSM-IV Disorders-I (SCID) [8]. However, since the SCID does not assess for the presence of ADHD, the interview version of the Wender-Reimherr Adult Attention Deficit Disorder Scale (WRAAS) [2] was used to provide a series of adult ADHD-focused questions. Subjects were rated on the WRAAS [2], Wender Utah Rating Scale [9] which inquires about childhood symptoms, Brown Attention Deficit Disorder Scale (Brown-ADD) for adults [10] and the Kellner Symptom Questionnaire [11]. Clinical investigators making the diagnosis were child and adult certified mental health professionals blind to results of the motion/attention tests.

Testing for capacity to sit-still and pay attention occurred during visits 1 and 2 (two tests per visit). Infrared motion capture occurred during performance of three different computerized attention tasks. Subjects also completed the Conners’ CPT-II attention task (Ver 5) without concomitant motion measures (as motion measures are not part of this test). The four attention tasks were administered in random order spread across the two test sessions.

The three different attention tasks with motion capture were the No-4’s cognitive control task, the Cued Response task and the Embedded Memory task. Stimuli for all tasks consisted on 4, 5, 8 and 16-pointed stars, which were presented at random screen positions. In the No-4’s cognitive control task subjects responded to all stars except those with 4 points. This is similar in principal to the CPT-II, in which subjects respond to all letters except X, and was a high target density task (~90% targets). In the Cued Response task subjects responded to 8-pointed stars, but only when a 5-pointed star immediately preceded them. This is similar to the AX-CPT [12], and was a low target density task (~20% targets). In the Embedded Memory task subjects were instructed to always respond to 8-pointed stars and to never respond to the 5-pointed stars. Further, 4- and 16-pointed stars became targets if the same type of star most recently preceded them. Hence, the first time a subject saw a 4-pointed star it was not a target, however any subsequent 4-pointed stars were targets until a 16-pointed star appeared. Similarly, the first 16-pointed star was not a target, however any subsequent 16-pointed stars were targets until a 4-pointed star appeared, etc. This was a moderate target density task (~50% targets).

Activity results were similar across all three motion capture/attention tasks. Results from only one of the tasks (the No-4’s cognitive control test that is available as part of the Quotient ADHD System) will be presented along with CPT-II results. The No-4’s task was selected for commercial development as its high target density facilitated analysis of attention state in sequential 30-second epochs [13]. The Cued Response Task and Embedded Memory Task were no better than the No-4’s task in distinguishing ADHD subjects from controls based on attention measures, and these tasks are no longer available.

### Assessments

#### Movement patterns

Two Qualisys ProLite infrared motion analysis cameras tracked head and lower extremity (shin and foot) movements (50 measures per second, 0.04 mm resolution). Foot movements were similar to shin movements, but were not captured as reliably – as foot markers were occasionally obscured from the camera’s view. Hence, we limited the analysis to head and shin measures. Movement patterns were analyzed using previously described procedures [5]. Measures include number of movements > 1 mm (microevents), total distance moved, area in which the movements occurred and two scaling exponents [14]. The spatial scaling exponent is a measure of the complexity of the movement path. The temporal scaling exponent provides a robust measure of relative activity versus inactivity [14]. The test also provides a composite measure of activity (*activity severity composite*), designed to be a treatment responsive metric of hyperactivity.

#### No-4’s cognitive control task

Stimuli consisted of 16-, 8-, 5 -and 4-pointed stars presented singly at random screen positions for 240 msec with a variable interstimulus interval (ISI) (mean 2500 msec). Subjects were instructed to press the spacebar for all stars except for those with 4 points. Ninety-percent of the stimuli were targets. Reported measures were overall accuracy, errors of omission (EOM), errors of commission (EOC), correct response latency, standard deviation of response latency, coefficient of variation (COV) in response latency, and measures of fluctuation in attentional state [13]. For this assessment the task was divided into 30-second epochs and calculations were made using Fisher’s Linear Discriminant Function as to whether the subject was either fully attentive, distracted, responding impulsively, randomly or was disengaged (minimally responsive) during each epoch. In addition, the task provided a composite index of distraction (*distraction severity composite*), designed to be a treatment responsive metric of inattention, and a *discriminative index* that combined both activity and attention parameters and was optimized using logistic regression to distinguish adolescent and adult subjects with ADHD from controls. The most important predictors in the logistic regression were leg measures, head measures, attention shifts and errors of omission.

#### Conners’ CPT-II

Stimuli consisted of letters presented at the center of the computer screen for 250 msec with an ISI of 1000, 2000 or 4000 msec (3 levels). The test consisted of 6 blocks made up of 3 sub-blocks – one sub-block for each ISI. Subjects were instructed to press the spacebar for all letters except X. Reported measures were EOM, EOC, reaction time (RT) for correct responses, standard error in correct RT, variability in RT across sub-blocks, d-prime (capacity to distinguish targets from non-targets), beta (response bias), perseveration (RTs less than 100 msec), and measures of change in correct RT and RT standard error across blocks and ISI levels. The Connor’s CPT-II provides a *confidence index* composite, based on discriminant analysis, which indicates the likelihood that an individual has an actual problem with attention. The most important predictors in the discriminant function were percent omissions, gender, age, beta, and RT across ISI.

### Data analysis

The aim of statistical analyses was to first ascertain whether there were differences between subjects with ADHD and controls in objective measures of activity, and if so, to test the hypothesis that attention measures were more significant and better able to discriminate between groups than objective measures of activity. Four different approaches, of increasing sophistication and complexity, were used to test this hypothesis. First, analysis of covariance (ANCOVA) was used to test for group differences in the individual measures of activity and attention. Effect size and area under the Receiver Operating Characteristic curve (ROC-AUC) were calculated for each covariate adjusted measure to provide a basis for comparison. Second, ROC analysis was used to compare the composite indices provided by each test (*activity severity composite, distraction severity composite, discriminative index and CPT-II confidence index*) to discriminate between ADHD subjects and controls in the sample. Third, Random forest regression was used as a novel analytic strategy to indicate the relative importance of each measure in discriminating the present sample of subjects with ADHD from controls. This technique is used in data mining and provides new measures of variable importance that are effective in delineating the most meaningful variables even in the presence of multicollinearity. Fourth, modeling techniques with cross-validation were used to assess the capacity of activity versus attention measures to discriminate subjects with ADHD from controls in a way that would likely generalize beyond the studied sample. Statistical analyses were conducted using R [15], and are detailed below.

ANCOVA was used to assess main effects of group, age and sex on the dependent variables. Motion analysis data were also adjusted to account for occasional lost frames or reflections (mean loss - 18%). We used the false discovery rate method of Benjamini and Hochberg [16] to minimize the risk of type I errors in the use of multiple ANCOVA tests in the assessment of between group, age and gender differences within each measurement category. Hence, q values are presented instead of p values to denote the probability value after adjustment for the number of comparisons within each category. Group mean values (with 95% confidence intervals) and effect size measures were calculated on covariate adjusted data, provided that the dependent variable was significantly influenced by the covariate (unadjusted p < 0.05). Effect size differences were indicated by Cohen’s d’ [17]. Confidence intervals for d’ were calculated using bootstrapping (1,000 estimates with resampling). The ROC-AUC was calculated for each covariate adjusted measure. An ROC curve is a plot of sensitivity versus 1 – specificity, and the area under the curve provides the best single indicator of the discriminative capacity of a test [18]. A perfectly accurate test has an ROC-AUC = 1.0. A test that is no better than chance has an ROC-AUC = 0.5. The ROC-AUC was estimated by the trapezoidal method and confidence intervals were estimated by the DeLong method (R package *DiagnosisMed 0.2.3*).

DeLong’s test for two correlated ROC curves [19] was used to assess the statistical differences between ROC curves for the activity severity and distraction severity composites, the discriminative index and the CPT-II confidence index. If activity has faded as a discriminant feature in adults with ADHD then the ROC-AUC for the activity severity composite should be inferior to the ROC-AUC for the distraction severity composite or the CPT-II confidence index.

Correlation coefficients with confidence intervals were used to explore the relationship between composite indices of activity and attention and ratings of executive function on the Brown ADD scale.

#### Random forest regression and classification

Random forest regression (R package *RandomForest 4.5-34*) was used to indicate the relative importance of each measure in discriminating subjects with ADHD from controls. This technique is used in data mining where the goal is to identify the most important subset of variables in a large collection as, for example, in microarray studies. Random forest regression was developed by Breiman [20] as an extension of the decision tree approach. It is a form of ‘ensemble learning’ in which a very large number of small unpruned decision trees are generated and their results aggregated. This technique performs very well compared to many other classifiers, including discriminant analysis, support vector machines and neural networks [21], provided that predictor variables are similar in their scale of measurement or number of categories [22].

Random forest classification indicates importance in two ways. One is a measure of how much the diagnostic accuracy of the forest is decreased by permutation (effective randomization and elimination) of a given predictor variable. The more the permutation of a variable degrades accuracy the greater the importance of the variable. The second measure of importance is the mean decrease in the Gini coefficient following permutation of a variable. The Gini coefficient is a measure of the inequality of a distribution. The better the nodes were at splitting the sample the higher the Gini coefficient. For this analysis 10,000 trees were generated with 4 variables tried per split. Results were compared with an alternative technique to minimize potential bias (*cforest* in R package *party*[22]). If hyperactivity has faded as a discriminative feature of adult ADHD then activity measures should be less important features of the random classification forest than attention measures.

#### Comparison of Activity and Attention Measures using Predictive Modeling

The question whether activity measures were less effective than attention measures in discriminating subjects with ADHD from controls was also assessed using predictive modeling techniques (R package *caret*[23]). A series of cross-validated models were generated using conventional (i.e., linear discriminant analysis, logistic regression, general linear models) and novel statistical approaches (i.e., neural networks, support vector machines, random forests) for completeness. Random forest, as described above, is a form of “ensemble learning” in which the elements are decision trees that branch based on the predictor variables to designate subjects as cases or controls. Neural networks are non-linear statistical data machine learning tools that use hidden layers of connections to model the relationship between inputs (predictor variables) and output (classification). Similarly, support vector machines represent a third approach to machine learning, in which the learning set of data points are mapped into a higher dimensional space via a kernel function, and a hyperplane identified that provides the greatest separation between points of different class (ADHD, control).

Each statistical model was generated using results from 75% of the subjects to build the model, which was then assessed for predictive accuracy on the remaining 25%. This process was repeated 200 times (5000 times for Random Forests) on different random splits of the data and the results average across all repetitions to yield a cross-validated estimate of the predictive discriminant ability of the measures that would likely generalize to new cases [23]. Variables included in each model were the subject’s age and gender and then either the 12 measures of attention task performance, or the 12 measures of head and lower extremity movements. Models based on attention measures were compared to models based on activity measures for accuracy, kappa (concordance with clinical diagnosis) and ROC-AUC. If activity has ceased to be an important discriminative feature between adults with and without ADHD then models based on activity measures should be inferior to models based on attention measures.

## Results

The ADHD group consisted of 40 subjects (23M/17F) with a mean age of 35 ± 10 years. Only 2 of the subjects were receiving pharmacological treatment at the time of recruitment. Control subjects included 60 adults (28M/32F) with a mean age of 29 ± 9 years (t = −3.06, df = 76.7, p = 0.003, Welch Two-Sample t-test). Subjects with ADHD had, on average, 7.0 ± 1.7 DSM-IV defined symptoms of inattention and 5.9 ± 2.5 symptoms of hyperactivity-impulsivity. In contrast, controls had, on average, less than one symptom of inattention or hyperactivity-impulsivity. On the WRAAS [2] subjects with ADHD differed most strongly from controls in ratings of attention difficulties and disorganization followed by ratings of impulsivity and finally ratings of hyperactivity. WRAAS ratings of inattention and disorganization discriminated ADHD subjects from controls more accurately than WRAAS ratings of hyperactivity (Inattention vs Hyperactivity: Z= 2.73, p = 0.006; Disorganization vs Hyperactivity Z=1.96, p < 0.05). Overall, 17 (9m/8f; 42.5%) subjects in the ADHD group were classified as predominantly inattentive. Subjects with ADHD did not differ from healthy adults in ratings of depression, anxiety, somatization or anger-hostility.

**Figure 1:**
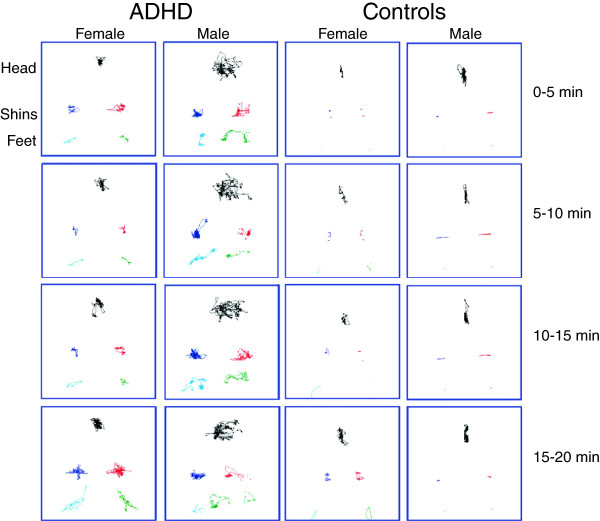
**Infrared motion analysis patterns tracing the movement path of markers attached to the head, left and right shins and ankles of four representative subjects in 5-minute blocks over the course of the 20-minute cognitive control task.** Subjects included in the figure were males and females from each group whose activity measures were closest to the ADHD and control group means.

Characteristic movement patterns of male and female subjects with and without ADHD are illustrated in Figure 1. Subjects with ADHD had a much greater rate and range of movements during the task. Almost all movement parameters revealed large effect sizes (Table 1). For example, subjects with ADHD moved their head through a 2.2-fold greater area (Cohen’s d’ = 0.98) and their shins through a 3.6-fold greater area (d’ = 1.05) than healthy adults. Spatial complexity was the most discriminative head movement measures based on the area under the Receiver Operating Characteristic Curve (ROC-AUC = 0.85). The discriminative abilities of all lower extremity measures were essentially equal (ROC-AUC = 0.87–0.88). However, the temporal scaling measure had the largest effect size (d’ = 1.6). Age and gender exerted no significant effects on these activity parameters, and there were no group x gender interactions that exceeded the false discovery rate.

**Table 1:** Differences between healthy controls and adults with ADHD on head and lower extremity movements

**Measures**	**Controls mean**	**ADHDmean**	ANCOVA* Group F	**Effect Size Cohens d'**	**ROC Area**
	**[95% CI]**	**[95% CI]**	**†q value**	**[95% CI]**	**[95% CI]**
*Head Movements*					
Immobility duration (sec)	0.42	0.18	14.97	0.85	0.77
	[0.33–0.50]	[0.16–0.21]	p<0.0003	[0.71–1.21]	[0.68–0.86]
Microevents	1283	2255	19.36	1.00	0.79
	[1055–1511]	[1907–2603]	p<0.00004	[0.64–1.34]	[0.70–0.88]
Displacement (m)	2.25	3.94	11.65	0.73	0.79
	[1.62–2.89]	[3.28–4.59]	p<0.001	[0.31–1.35]	[0.70–0.88]
Area (cm2)	65.02	145.6	24.09	0.98	0.82
	[45.38–84.66]	[116.6–174.7]	p<10^-5^	[0.56–1.50]	[0.74–0.90]
Spatial Complexity	1.31	1.11	25.71	1.08	0.85
	[1.25–1.37]	[1.09–1.13]	p<10^-5^	[0.92–1.52]	[0.78–0.93]
Temporal Scaling	0.41	0.63	12.58	0.83	0.72
	[0.34–0.49]	[0.56–0.71]	p<0.0007	[0.44–1.25]	[0.63–0.82]
*Shin Movements*					
Immobility duration (sec)	4.84	0.74	29.62	1.10	0.88
	[3.60–6.08]	[0.53–0.94]	p<10^-5^	[0.99–1.50]	[0.81–0.95]
Microevents	250.7	1048.4	23.69	1.01	0.88
	[149.6–351.8]	[680.4–1416.5]	p<10^-5^	[0.71–1.27]	[0.81–0.94]
Displacement (m)	0.44	1.93	22.42	0.96	0.88
	[0.21–0.67]	[1.23–2.64]	p<0.00002	[0.64–1.22]	[0.81–0.95]
Area (cm2)	12.65	45.36	27.09	1.05	0.88
	[6.32–18.99]	[32.79–57.92]	p<10^-5^	[0.62–1.60]	[0.82–0.95]
Spatial Complexity	2.22	1.33	44.44	1.45	0.87
	[2.02–2.42]	[1.26–1.40]	p<10^-7^	[1.26–1.95]	[0.80–0.94]
Temporal Scaling	0.07	0.49	57.25	1.60	0.88
	[−0.001–0.14]	[0.42–0.57]	p<10^-9^	[1.26–2.11]	[0.81–0.95]

*Main effect of group. Effects of Age and Gender were not significant for any measure after correction for multiple comparisons.

†q value = p value adjusted for multiple comparisons using the False Discovery Rate method of Benjamini and Hochberg.

The most robust differences in attention on the No-4’s test were on percent time spent fully attentive (d’ = 0.87), accuracy (d’ = 0.76) and response latency coefficient of variation (COV, d’ = 0.76). The remaining parameters, when significant, were associated with moderate to small effect sizes (Table 2). Measures of percent time fully attentive and response latency COV distinguished ADHD subjects from controls with ROC-AUC of 0.73. None of the potential age or gender effects survived correction for multiple comparisons.

**Table 2:** Differences between healthy controls and adults with ADHD on No4's attention test

**Measures**	**Controls mean**	**ADHD mean**	ANCOVA* Group F	**Effect Size Cohens d'**	**ROC Area**
	**[95% CI]**	**[95% CI]**	**†q value**	**[95% CI]**	**[95% CI]**
Accuracy (%)	97.46	95.76	16.83	0.76	0.71
	[97.00–97.93]	[94.87–96.65]	p<0.0006	[0.38–1.13]	[0.62–0.79]
Errors of Omission	0.54	1.5	9.22	0.67	0.71
	[0.33–0.76]	[0.86–2.13]	p<0.008	[0.32–0.94]	[0.65–0.77]
Errors of Comission	20.48	30.71	11.23	0.64	0.67
	[16.87–24.09]	[24.84–36.58]	p<0.004	[0.24–1.08]	[0.56–0.78]
Latency (msec)	482.9	481.6	0.11	0.02	0.52
	[467.1–498.7]	[459.5–503.7]	p>0.7	[−0.38–0.45]	[0.40–0.64]
Latency SD	97.93	119.4	8.75	0.69	0.70
	[90.38–105.5]	[108.6–130.1]	p<0.008	[0.28–1.12]	[0.60–0.80]
Latency COV	20.37	24.32	13.43	0.76	0.73
	[19.06–21.67]	[22.58–26.07]	p<0.002	[0.38–1.16]	[0.66–0.80]
Attention Shift	13.95	15.22	2.27	0.27	0.58
	[12.62–15.28]	[13.93–16.52]	p>0.1	[−0.15–0.67]	[0.50–0.66]
Attentive (% time)	60%	42%	19.28	0.87	0.73
	[55–65%]	[35–49%]	p<0.0004	[0.49–1.33]	[0.62–0.83]
Distracted (% time)	16%	25%	4.78	0.54	0.65
	[13–20%]	[19–31%]	p<0.05	[0.10–0.94]	[0.55–0.75]
Impulsive (% time)	23%	32%	7.41	0.51	0.62
	[19–27%]	[26–39%]	p<0.02	[0.10–0.93]	[0.51–0.74]
Random (% time)	0.22%	0.76%	1.92	0.36	0.54
	[0.0–0.4%]	[0.1–1.4%]	p>0.1	[−0.08–0.63]	[0.48–0.60]
Minimal (% time)	0.19%	0.99%	4.09	0.44	0.56
	[0.0–0.5%]	[0.2–1.8%]	p<0.07	[−0.02–0.70]	[0.50–0.62]

*Main effect of group. Effects of Age and Gender were not significant for any measure after correction for multiple comparisons.

†q value = p value adjusted for multiple comparisons using the False Discovery Rate method of Benjamini and Hochberg.

Only four of the Conners’ CPT-II measures showed a significant main effect of group after correcting for multiple comparisons. Differences between subjects with ADHD and controls on these few measures were associated with moderate to small effect sizes (Table 3). The most significant differences observed were in errors of commission (EOC, d’ = 0.60) and in the standard error of reaction time (d’ = 0.55). The most discriminative single parameter was EOC, with an ROC-AUC = 0.67. There were no significant differences in these measures by age or gender after correction for multiple comparisons.

**Table 3:** Differences between healthy controls and adults with ADHD on Conners' CPT-II attention test

**Measures**	**Controls mean**	**ADHD mean**	ANCOVA* Group F	**Effect Size Cohens d’**	**ROC Area**
	**[95% CI]**	**[95% CI]**	**†q value**	**[95% CI]**	**[95% CI]**
Errors of Omission	1.95	1.79	0.15	0.05	0.47
	[0.91-2.98]	[0.93-2.65]	p>0.8	[-0.41-0.39]	[0.45-0.60]
Errors of Comission	8.70	12.26	8.30	0.60	0.67
	[7.26-10.15]	[10.11-14.42]	p<0.03	0.17-1.09	0.58-0.77
Correct Reaction Time (RT)	385.2	389.3	0.01	0.07	0.52
	[370.5-399.9]	[369.8-408.9]	p>0.9	[-0.34-0.51]	[0.40-0.64]
RT Standard Error (RT SE)	5.31	6.35	8.13	0.55	0.63
	[4.86-5.77]	[5.65-7.05]	p<0.03	[0.08-0.86]	0.53-0.74
Variability in RT by Blocks	6.76	8.65	7.55	0.48	0.63
	[5.87-7.65]	[7.14-10.16]	p<0.03	[0.08-0.86]	[0.53-0.74]
Discriminability (d prime)	0.96	0.74	3.95	0.49	0.66
	[0.84-1.07]	[0.61-0.88]	p<0.09	[0.09-0.96]	[0.58-0.75]
Response Bias (beta)	0.91	0.68	1.45	0.23	0.57
	[0.62-1.20]	[0.45-0.92]	p<0.3	[-0.17-0.57]	[0.58-0.75]
Perseverative errors	0.19	0.71	7.07	0.46	0.61
	[0.03-0.36]	[0.19-1.23]	p<0.03	[0.11-0.76]	[0.56-0.66]
Change in RT by Blocks	0	0	0.02	0.08	0.51
	[-0.007-0.007]	[-0.012-0.017]	p<0.8	[0.36-0.52]	[0.46-0.56]
Change in RT SE by Blocks	0	0	0.02	0.02	0.5
	[-0.021-0.014]	[-0.034-0.023]	p<0.9	[-0.04-0.46]	[0.45-0.56]
Change in RT by ISI	0.05	0.07	4.5	0.43	0.61
	[0.035-0.063]	[0.056-0.086]	p<0.08	[0.05-0.90]	[0.58-0.64]
Change in RT SE by ISI	0.01	0.06	4.41	0.39	0.6
	[-0.011-0.039]	[0.015-0.095]	p<0.08	[-0.02-0.80]	[0.55-0.66]

Main effect of group. Effects of Age and Gender were not significant for any measure after correction for multiple comparisons.

†q value = p value adjusted for multiple comparisons using the False Discovery Rate method of Benjamini and Hochberg.

**Figure 2:**
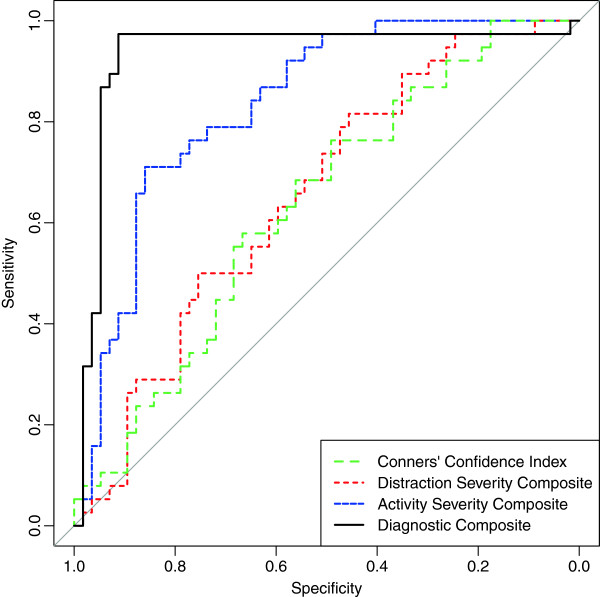
**Receiver operating characteristic curves for age and gender covaried composite measures of attention and activity.** Conners’ Confidence Index comes from the Conners CPT-II. Attention Severity, Activity Severity and Diagnostic Composites derived from the No-4’s cognitive control task and infrared motion analysis.

Figure 2 shows the ROC curves for composite measures on the 95 subjects tested on the Connors’ CPT and No-4’s test with motion measures. The distraction severity composite on the Quotient ADHD Test was a weak discriminator with an ROC-AUC of 0.65 (95% CI 0.54–0.76). The Connors’ CPT-II confidence index was also a weak discriminator with an age and sex adjusted ROC-AUC of only 0.63 (95% CI 0.52–0.78). In contrast, the activity severity composite provided a greater degree of discriminative ability than either the distraction severity composite (Z = 2.90, p < 0.004) or the CPT-II confidence index (Z = 2.80, p < 0.006) with an ROC-AUC of 0.83 (95% CI 0.75–0.91). Finally, the discriminative index, which combines both activity and attention measures, was able to discriminate ADHD subjects from controls with an ROC-AUC of 0.96 (95% CI 0.93–0.99).

The greater discriminative capacity of activity versus attention measures was not limited to subjects with combined or predominantly hyperactive/impulsive subtypes. ROC-AUCs for discriminating predominantly inattentive subtype ADHD from controls were only 0.62 (95% CI 0.48–0.77) and 0.53 (95% CI 0.38–0.67) for the CPT-II confidence index and Quotient distraction severity composite, respectively. In contrast, the activity severity composite discriminated predominantly inattentive subtype ADHD from controls with ROC-AUC of 0.81 (95% CI 0.71–0.92). Combining both activity and attention measures into the discriminative index provided excellent separation with an ROC-AUC of 0.90 (95% CI 0.78–1.00).

**Figure 3:**
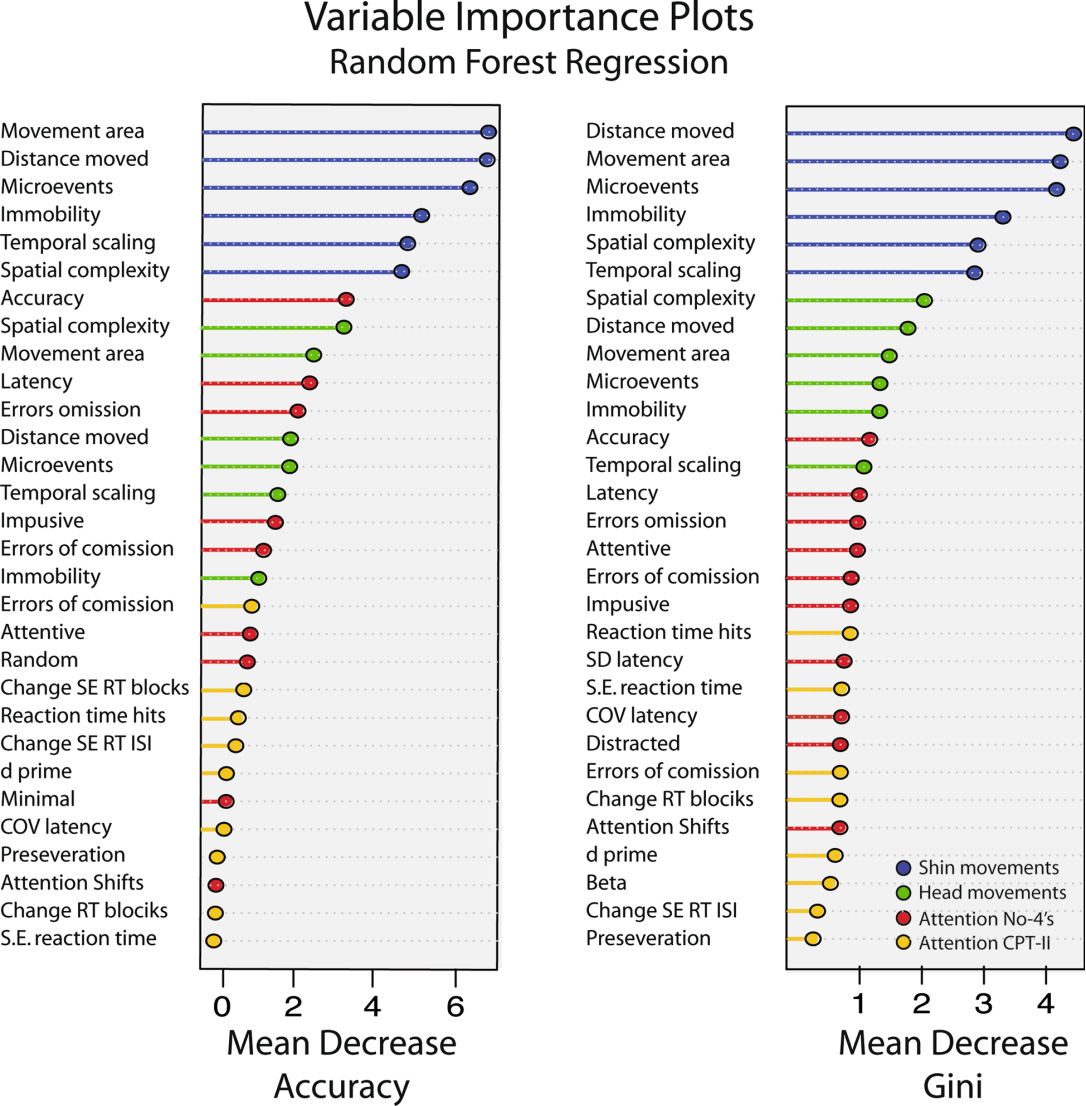
**Random forest regression analysis showing the relative importance of activity and attention measures in classifying subjects as ADHD or controls.** Variables are rank ordered by importance, which was determined in two ways. The left panel indicates importance by how much the permutation (effective elimination) of a given variable decreases the accuracy of the overall fit. The right panel indicates importance by how much the permutation of a variable attenuates the ability of the specific nodes in the random forest to accurately split the sample. Variables that are associated with the greatest decrease in accuracy or Gini coefficient following permutation are the most important.

Figure 3 shows the relative importance of each measure as assessed by random forest regression. Shin movements were clearly the most important discriminative variables followed by head movements based on either mean square error or Gini coefficient criteria. Attention measures were least important. Results were quite similar using *cforest* to generate conditional inference trees (*r* = 0.91).

Table 4 shows the relationship between composite measures of activity and inattention and self-report ratings of executive functions on the Brown ADD Scale. Impairments in executive functions in adults with ADHD correlate with problems in occupational performance [24], and disturbances in executive function are considered by some investigators to be the underlying problem in ADHD, with inattention, hyperactivity and impulsivity as byproducts [25,26]. If hyperactivity abates in adults with ADHD then objective measures of activity should correlate less strongly with executive function ratings than objective measures of inattention. All of the correlations were significant. The correlations between the various executive function ratings and either the distraction severity composite or the CPT-II confidence index fell along the border between small (*r* = 0.1 – 0.23) and moderate (*r* = 0.24 – 0.36) in effect size [27]. In contrast, correlations between executive function ratings and the activity severity constant were nearly two-fold greater and had a large effect size (*r* > 0.37). Including both activity and attention metrics into a discriminative index provided correlations coefficients of large size that explained between 27%-52% of the variance in the different executive function ratings.

**Table 4:** Correlation coefficients and 95% confidence intervals indicating relationships between Brown Attention Deficit Disorder executive function ratings and composite measures of activity and inattention

**Brown ADHD Ratings**	**CPT-II Confidence Index**	**Distraction Composite**	**Activity Composite**	**Discriminative Index**
Activation	0.25 [0.03–0.44]	0.23 [0.01–0.43]	0.51 [0.33–0.66]	0.66 [0.52–0.77]
Attention	0.23 [0.01–0.43]	0.28 [0.06–0.47]	0.54 [0.37–0.68]	0.72 [0.59–0.81]
Effort	0.33 [0.11–0.51]	0.28 [0.07–0.48]	0.51 [0.33–0.66]	0.60 [0.44–0.73]
Affective Control	0.23 [0.00–0.43]	0.23 [0.01–0.43]	0.40 [0.19–0.57]	0.52 [0.34–0.67]
Memory	0.28 [0.06–0.47]	0.26 [0.04–0.45]	0.49 [0.30–0.64]	0.62 [0.46–0.74]
TOTAL SCORE	0.28 [0.06–0.47]	0.28 [0.06–0.47]	0.54 [0.36–0.68]	0.68 [0.54–0.79]

Table 5 shows the comparative ability of cross-validated models using either linear discriminant, general linear models, logistic regression, neural networks, support vector machines, or random forests analytical techniques to predictively discriminate subjects with ADHD from controls. The accuracy of models using activity measures alone ranged from 0.766 – 0.842, depending on the underlying method. In contrast, models using just the No-4’s attention measures had accuracies that ranged from 0.650-0.720, and models based on CPT-II measures yielded accuracies between 0.624-0.709. Statistically, models based on activity measures alone were invariably superior to models based on attention measures (all p’s < 10^-15^) whether using accuracy, kappa or ROC-AUC as evaluative metrics.

**Table 5:** Comparison of predictive discriminatory accuracy of cross-validated models using measures of activity versus attention

**Criteria**	**Linear Discriminant Analysis**			**General Linear Model**			**Multinomial Logistic Regession**		
	**No 4's Attn**	**CPT-II Attn**	**Activity**	**No 4's Attn**	**CPT-II Attn**	**Activity**	**No 4's Attn**	**CPT-II Attn**	**Activity**
Accuracy	0.659	0.670	0.768^ab^	0.650	0.663	0.766^ab^	0.703	0.706	0.801^ab^
Kappa	0.257	0.281	0.505^ab^	0.240	0.269	0.504^ab^	0.360	0.360	0.583^ab^
ROC-AUC	0.671	0.712	0.821^ab^	0.661	0.692	0.800^ab^	0.722	0.729	0.839^ab^
Sensitivity	0.786	0.790	0.835^cd^	0.752	0.776	0.816^ef^	0.800	0.821	0.829
Specificity	0.462	0.482	0.665^ab^	0.491	0.486	0.687^ab^	0.552	0.528	0.758^ab^
**Criteria**	**Neural Network**			**Support Vector Machine**			**Random Forest Classification**		
	**No 4's Attn**	**CPT-II Attn**	**Activity**	**No 4's Attn**	**CPT-II Attn**	**Activity**	**No 4's Attn**	**CPT-II Attn**	**Activity**
Accuracy	0.659	0.677	0.809^ab^	0.720	0.709	0.817^ab^	0.663	0.624	0.842^ab^
Kappa	0.273	0.299	0.607^ab^	0.396	0.376	0.621^ab^	0.292	0.191	0.676^ab^
ROC-AUC	0.687	0.711	0.816^ab^	0.731	0.753	0.829^ab^	0.661	0.673	0.889^ab^
Sensitivity	0.742	0.769	0.801^ab^	0.811	0.793	0.807	0.753	0.775	0.828^ab^
Specificity	0.528	0.534	0.802^ab^	0.578	0.578	0.833^ab^	0.543	0.415	0.870^ab^

^a^Activity > No4's Attention p < 10^–15^, ^b^Activity > CPT-II Attention p < 10^–15^.

^c^Activity > No4's Attention p < 10^–6^, ^d^Activity > CPT-II Attention p < 10^–5^.

^e^Activity > No4's Attention p < 10^–8^, ^f^Activity > CPT-II Attention p < 0.0001.

## Discussion

Contrary to conventional wisdom, adults with ADHD manifest clear signs of hyperactivity on objective assessment. Interestingly, effect sizes for ADHD versus control differences in individual activity measures were, on average, 2-fold greater than effect sizes for differences for No-4’s attention measures and 3-fold greater than CPT-II attention measures. These results are similar to findings in children with ADHD [5,13,28]. Activity measures were superior to attention measures even when distinguishing predominantly inattentive subjects with ADHD from control subjects. Random forest classification provided additional confirmation that activity measures were considerably more important than attention measures in discriminating adults with ADHD from healthy controls in this sample. The superiority of activity measures over attention measures emerged when examining the ROC-AUC curves for the composite index scores and in all of the cross-validated predictive mathematical models. In short, objective data provides no support for the hypothesis that hyperactivity fades with age and becomes a less discriminatory feature of the disorder.

This observation was not likely an artifact of recruiting an abnormally hyperactive sample of adults with ADHD. Clinical ratings of these subjects on the WRAAS showed that activity differences were significantly less robust than ratings of attention, or disorganization, as has been observed in previous adult samples. Further, activity measures remained superior to attention measures in discriminating even predominantly inattentive subtype ADHD adults from controls. Objective measures of activity may differ from clinical impressions as subjective ratings of hyperactivity are skewed by the valence of the behavior, such that aggressive individuals are rated as hyperactive regardless of their actual activity levels [29]. The clinical impression that hyperactivity fades with age may actually be a reflection of fading levels of aggression rather than a normalization of capacity to sit still or to inhibit activity to low levels. Further, the signs or symptoms of hyperactivity used in clinical ratings and diagnostic criteria may simply be age inappropriate for adults. Draft criteria for DSM-5 include four additional symptoms of hyperactivity-impulsivity to better capture the phenomenon in adults.

There are a number of possible interpretations for the predominance of objective activity over attention measures. First, activity may be a more powerful discriminator because it can be measured more accurately. Activity is a physical property and motion analysis systems can track overt movements with precision. Attention is an internal state and is not directly assessed but rather inferred from a subject’s performance on a task.

Second, we may have underestimated the importance of attention by selecting suboptimal tasks, though we are not aware of any task that would have provided superior results. For example, the most robust differences between children with ADHD and controls on the stop signal delay task and Stroop have smaller mean effect sizes (i.e., 0.73 [30] and 0.58 [31], respectively) than what we observed in the present study. Meta analyses show that effect size differences for measures of executive function are of intermediate magnitude (d’ = 0.46 – 0.69) [32], with the largest reported differences in adults occurring on the Trails Making Test B (d’=0.73) [33]. Schweiger et al. [34] evaluated a computerized test battery in 28 male undergraduates with ADHD and 49 controls. The battery sampled a wide range of cognitive domains including verbal and non-verbal memory, executive function, visual spatial processing, information processing, motor speed and problem solving ability. Significant effect size measures ranged from 0.50 – 0.87. The greatest between-group differences emerged during an extended CPT that was very similar to the CPT-II and No-4’s test [34]. In short, computer measures of attention or executive function reported in all the studies we examined had effect size findings that were at best equivalent to, and in almost all instances inferior to, effect size differences we observed on the No-4’s test.

Third, attention deficits may simply not be as fundamental to the disorder as the name implies. Individuals with ADHD appear to have problems ‘paying attention’ when tasks are boring or unrewarding, but research shows that they can usually perform as well as controls on tasks that are engaging or provide sufficient incentives [35-39]. The evidence suggests that their attentional abilities are relatively intact, and their performance difficulties are more related to problems with motivation [35,36,39], reward processing [38] or inhibitory control [40]. Interestingly, ADHD is one of the few psychiatric disorders whose closest equivalent in the International Classification of Disease has a completely different name; i.e., *Hyperkinetic Disorder* – emphasizing a very different feature of the disorder.

The fourth possibility, which follows logically, is that the ability to inhibit motor activity to low levels during a sustained attention task may be a particularly good index of the primary problem present in individuals with ADHD. Barkley’s theoretical model postulates that the essential impairment in ADHD is a deficit involving response inhibition [40]. This deficit leads, in turn, to an array of problems with executive functions that depend on behavioral inhibition for their execution [40]. Neurobiologically, the capacity to inhibit both voluntary and spontaneous motor activity depends on prefrontal corticostriatal circuits [41-48]. Hence, it may be the case that the capacity to inhibit voluntary responses and the capacity to inhibit spontaneous movements are closely allied. If so, measuring the latter may provide a meaningful index of behavioral inhibition. This possibility is supported by the observation (consonant with Barkley’s theory) that the activity severity composite correlated strongly with multiple domains of impaired executive function on the Brown ADD Scale, and explained about four-fold more of the variance in executive function ratings than either of the attention composites. In short, the strong association between impairments in executive function and objective measures of activity is consistent with the hypothesis that these phenomena are interrelated neurobiologically.

In this regard we have reported a highly significant association between objective measures of hyperactivity and T2-relaxation time (an indirect index of diminished resting cerebral blood volume) in the putamen [6], which was further strengthened when T2-relaxation time in the right dorsolateral prefrontal cortex was also taken into account [49]. Jucaite et al. [7] also reported a strong correlation between D2 receptor binding in the right caudate nucleus and motion analysis measures of head movements. Conversely, recent studies have also reported strong associations between measures of caudate and putamen volume, functional activity, connectivity or dopamine release and executive functions in typically developing children [50], healthy adults [51-53], elderly adults [54] and patients with schizophrenia and bipolar disorder [55]. There is, in short, increasing recognition that tonic release of dopamine into the caudate and putamen, in addition to regulating motor activity, also acts to focus and filter non-motor activities such as working memory, implicit learning, decision making, and planning [56]. Hence, there are compelling reasons to link inhibitory motor control and executive functions, and to envision that a single abnormality may frequently be responsible for deficits in both domains.

These present findings show that tests of attention by themselves perform suboptimally as diagnostic aids for ADHD. This evidence is consonant with previous reports in adults and children. For example, Epstein, Conners’ and colleagues observed that the CPT-II discriminated adults with ADHD from controls with only 55% sensitivity and 76.4% specificity [57]. Similarly, Edwards et al. [58] reported that the CPT-II confidence index had a maximal ROC-AUC of only 0.64 in classifying children with ADHD. We observed an ROC-AUC of 0.63 for classifying adults with ADHD.

There are a number of design differences between the CPT-II and the No-4’s cognitive control task, such as the use of relatively large geometric shapes presented at random screen positions versus the presentation of smaller letters at the same fixed center point. The CPT-II emphasized signal detection theory and regression measures of performance change across blocks or interstimulus intervals. The No-4’s task emphasized an analysis based on fluctuations in attention state [13]. Random forest regression indicated that 5 or 6 of the No-4’s measures were more important discriminators than any of the CPT-II measures based on mean accuracy or Gini criteria. However, in the end the differences were minor. Neither the CPT-II nor the No-4’s test (by itself) could discriminate between adults with ADHD and healthy controls with acceptable levels of accuracy.

In contrast, measurements of motor activity alone in the present study had the predictive capacity to differentiate a mixed group of ADHD adults from a control group with up to 83% sensitivity and 87% specificity (Table 5), and in a recently published study discriminated 62 combined subtype children with ADHD from 62 controls with up to 100% accuracy [28]. Combining activity and attention measures in the present study provided excellent discrimination of subjects with ADHD versus controls (ROC-AUC = 0.96). While we do not believe that these measures can substitute for a comprehensive clinical assessment, we do believe that they may aid in diagnosis by providing objective information, and precisely measured target symptoms for gauging response to treatment.

The main limitations of this study are moderate sample size and the selection of adults with ADHD without comorbidity. Further studies will need to assess whether these findings can be replicated, extended to subjects with comorbid disorders, and tested for their capacity to differentiate adults with ADHD from subjects with other disorders that alter activity or impair attention.

## Conclusions

Although it is widely believed that hyperactivity in individuals with ADHD abates with age we found, using objective measures, that adults with ADHD moved their head and legs to a markedly greater degree than healthy controls during performance of a computerized attention test. Indeed, activity measures were far better at discriminating adults with ADHD from controls than computerized measures of inattention and impulsivity. This was true for both males and females with ADHD and for ADHD subjects diagnosed with the predominantly inattentive subtype. These findings are very similar to results previously reported in children using the same technology (infrared motion analysis) [5,13]. In addition, objective measures of activity correlated to a substantial degree with ratings of impaired executive function, and explained about four-fold more of the variance in these ratings than attention measures. These findings are important as they call into question the hypothesis that neuromaturational changes occur in individuals with ADHD that preferentially improve motor activity. Instead these findings suggest that a deficient capacity to inhibit motor activity to low levels remains a defining feature of the disorder, and that this deficit is closely linked to reported difficulties in executive control. The present findings also confirm that computerized measures of inattention by themselves have only a very limited ability to identify subjects with ADHD (ROC area 0.63 – 0.65). In contrast, motor activity measures using infrared motion analysis had a good capacity to discriminate adults with ADHD from healthy controls (ROC area 0.83), and when combined with attention measures had an excellent ability (ROC area 0.96). These findings also suggest that clinical ratings of hyperactivity, which attenuate with age, may do so because they become increasingly less apropos outside of childhood. Hence, the evaluation of adults with ADHD and monitoring of response to treatment may be enhanced by either the incorporation of objective measures of motor activity, or by the creation of more developmentally appropriate rating scales and criteria.

## Abbreviations

ADHD: Attention deficit hyperactivity disorder; AUC: Area under the curve; Brown-ADD: Brown attention deficit disorder scale; CI: Confidence interval; COV: Coefficient of variation; EOC: Errors of commission; EOM: Errors of omission; ISI: Interstimulus interval; ROC: Receiver operating characteristic; RT: Reaction time; WRAAS: Wender-Reimherr adult ADHD scale.

## Competing interests

The Quotient™ technology used in this study is owned by McLean Hospital and licensed to BioBehavioral Diagnostic Company (BioBDx). Dr. Teicher is the inventor on a number of patents related to this technology and receives a portion of the royalties paid to McLean from the use of Quotient™, in compliance with guidelines established by Harvard Medical School to minimize conflict of interest in clinical research. Dr. Teicher has no equity interest in BioBDx and holds no management position. Dr. Teicher has been reimbursed by BioBDx for travel expenses incurred to present results of research on Quotient™, has received funding from BioBDx for research relating to Quotient™, and receives consulting fees that fall within the *de minimis* guidelines established by Harvard Medical School and Partners Health Care. The Committee on Conflicts of Interest at Partners Health Care provided specific guidelines, including independent statistical analyses, to manage potential conflicts relating to this study. Dr. Teicher also received research support, as a component of an NIMH SBIR award, from Ambulatory Monitoring Inc., to investigate use of a feedback actigraph in treatment of children with ADHD. He previously received ADHD-related research funding from Copley Pharmaceuticals, Repligen and OPTAx Systems Inc., the latter also paid consulting fees. He has received funding for depression research from The LiteBook Company and CNS Response. Dr. Teicher has not received any consulting or speaking fees from pharmaceutical companies within the last five years.

Dr. Fourligas was a part-time employee of BioBDx from January 2007 through February 2007, a full-time employee from March, 2007 to November, 2008 and has continued to work for the company on a limited part-time basis. He has been granted options to purchase shares under the company's Stock Incentive Plan for services to the company. A portion of these shares have become exercisable.

Drs. Polcari, Vitaliano and Navalta have no conflicts to declare.

## Authors’ contributions

MHT conceived the study, participated in its design and coordination, performed the statistical analyses and drafted the manuscript. AP participated in the design and coordination of the study, conducted the diagnostic assessments, and provided critical comments on the drafts. NF participated in the design of the study, provided technical expertise on the software and hardware for data capture and provided critical comments on the drafts. GV helped to draft the manuscript, developed the literature review and reviewed the statistical results. CN participated in the design and coordination of the study, conducted the diagnostic assessments, and provided critical comments on the drafts. All authors read and approved the final manuscript.

## Pre-publication history

The pre-publication history for this paper can be accessed here:

http://www.biomedcentral.com/1471-244X/12/190/prepub
